# Development of Smart Bilayer Alginate/Agar Film Containing Anthocyanin and Catechin-Lysozyme

**DOI:** 10.3390/polym14225042

**Published:** 2022-11-21

**Authors:** Orapan Romruen, Pimonpan Kaewprachu, Thomas Karbowiak, Saroat Rawdkuen

**Affiliations:** 1 Food Science and Technology Program, School of Agro-Industry, Mae Fah Luang University, Chiang Rai 57100, Thailand; 2 College of Maritime Studies and Management, Chiang Mai University, Samut Sakhon 74000, Thailand; 3 Cluster of Innovative Food and Agro-Industry, Chiang Mai University, Chiang Mai 50100, Thailand; 4 Agrosup Dijon, UMR PAM A 02.102, Université de Bourgogne Franche-Comté, Esplanade Erasme, 21000 Dijon, France; 5 Unit of Innovative Food Packaging and Biomaterials, School of Agro-Industry, Mae Fah Luang University, Chiang Rai 57100, Thailand

**Keywords:** anthocyanin, antioxidant, antimicrobial, smart bilayer film, cellulose nanosphere, pH indicator, reduce waste generation, food loss

## Abstract

Smart packaging can provide real-time information about changes in food quality and impart a protective effect to the food product by using active agents. This study aimed to develop a smart bilayer film (alginate/agar) with a cellulose nanosphere (CNs) from corncob. The bilayer films were prepared using 1.5% (*w*/*w*) sodium alginate with 0.25% (*w*/*v*) butterfly pea extract incorporated (indicator layer) and 2% (*w*/*w*) agar containing 0.5% (*w*/*v*) catechin–lysozyme (ratio 1:1) (active layer). The CNs were incorporated into the alginate layer at different concentrations (0, 5, 10, 20, and 30% *w*/*w*-based film) in order to improve the film’s properties. The thickness of smart bilayer film dramatically increased with the increase of CNs concentration. The inclusion of CNs reduced the transparency and elongation at break of the smart bilayer film while increasing its tensile strength (*p* < 0.05). The integration of CNs did not significantly affect the solubility and water vapor permeability of the smart bilayer film (*p* > 0.05). The smart bilayer film displayed a blue film with a glossy (without CNs) or matte surface (with CNs). The developed bilayer film shows excellent pH sensitivity, changing color at a wide range of pHs, and has a good response to ammonia and acetic acid gases. The film possesses exceptional antimicrobial and antioxidant activities. The integration of CNs did not influence the antibacterial activity of the film, despite the presence of a higher level of DPPH in film containing CNs. The smart bilayer film was effectively used to monitor shrimp freshness. These findings imply that smart bilayer films with and without CNs facilitate food safety and increase food shelf life by monitoring food quality.

## 1. Introduction

Recently, new food packaging concepts have emerged in response consumers’ increased concern about the safety and quality of food products [1]. Detecting food freshness now requires specialized testing equipment, which is out of reach for most consumers. As a result, there is an essential need for a simple and practical approach to monitoring changes in food freshness. “Smart packaging” refers to active and intelligent packaging concepts that provide precise information regarding food conditions, thereby preserving the food product with antioxidant and antibacterial chemicals [2,3]. When protein-rich foods degrade, they release volatile amines, causing apparent color changes in pH-sensitive indicators [4]. Despite the effectiveness of synthetic dyes, they are not suited for food applications due to their toxicity, which endangers the health of consumers and the environment [5]. Many natural colorants, including anthocyanin, curcumin, and betalains, have been used to produce freshness indicator films in substitute for synthetic dyes [6,7]. Anthocyanin is a non-toxic natural pH-sensitive compound that has recently been used in laboratories to produce colorimetric indicator films [8]. Many sources of anthocyanin have been reported to be useful as an indicator for the preparation of freshness indicator films, such as red cabbage [9], sweet purple potato [10], blueberry [11], and butterfly pea [12]. Due to the potential of anthocyanin from butterfly pea as a natural colorant and antioxidant, it has been exploited in culinary and medical applications [13,14]. Butterfly pea anthocyanin is light and temperature stable, and it exhibits remarkable color changes at neutral, acidic, and basic conditions, making it a perfect candidate for color indicator films [15].

In order to achieve the purposes of smart packaging, the packaging should assist in prolonging or extending a food product’s shelf life. As a result, films typically use anti-microbials to improve their functional qualities and prolong the shelf life of food. Tea catechins contain many biological properties, including antioxidant, antimicrobial, and antifungal properties [16]. Lysozyme is an antibacterial protein in several biological tissues, cells, and fluids [17]. In recent years, edible food packaging films/coatings containing lysozyme have attracted the interest of many scientists [18]. According to Rawdkuen, et al. [19], the combination of catechin and lysozyme enhances antibacterial activity over either catechin or lysozyme alone.

New technologies for producing biodegradable packaging from renewable polymers have been developed due to the rising customer demand for eco-friendly and high-quality products generated from natural resources [20,21,22]. Agar is a polysaccharide derived from the Rhodophyceae family of marine red algae [23]. As agar films are transparent, biodegradable, and physiologically inert, they can readily interact with various bioactive chemicals [24,25]. However, pure agar film has restricted applicability due to its weak thermal stability, high water sensitivity, and water vapor permeability [26]. One of the most common strategies used to circumvent these limitations is the combination of agar with other substances, such as biopolymers, hydrophobic compounds, plasticizers, and nanoparticles. Sodium alginate (SA), a naturally occurring linear polysaccharide in brown algae, and which contains β-D-mannuronic acid and α-L-guluronic acid, is abundant and safe. Its strong water solubility and film-forming characteristics make it popular in the pharmaceutical and food sectors [27].

Bilayer film is a method for improving the properties of polymer films by combining materials with different properties in order to produce a two-layer film [28]. The properties of bilayer films are influenced by several factors, including preparation techniques, material types, and layer thickness ratios [29,30]. “Casting” is a broad term used for the two-step process of manufacturing bilayer film. Pouring and drying the first solution results in the formation of the first layer of film. As a result, the formed film is covered with the second film-forming solution, resulting in a bilayer film [31].

Nanocelluloses have recently received significant interest among the many nanomaterials for polymer reinforcement [32,33]. Wang, et al. [34] synthesized cellulose nanowhiskers from mulberry pulp and integrated them into alginate films. Their results indicated that the tensile strength of alginate films increased by 25%, although their water vapor permeability was unaffected. Roy, et al. [35] prepared smart films by incorporating cellulose nanocrystals (CNC) obtained from onion peel and shikonin into CMC/agar film. The addition of CNC considerably enhanced the mechanical, barrier, and optical properties of the CMC/agar film. Salim, et al. [36] discovered that the addition of CNC from pea pods improved the mechanical strength and thermal stability of chitosan films. Cellulose nanospheres (CNs) are a new form of nanocellulose with a spherical shape and nanoscale size [37]. The crystallinity and thermal stability of the polymer matrix can be enhanced by adding CNs, as these have a higher potential than rod-shaped nanocellulose at the same concentration [38]. According to our previous study (Romruen, et al. [39]), the utilization of cellulose nanosphere from different agricultural byproducts. Corncob CNs exhibit better properties than other materials. Therefore, it was used as a reinforcing material in order to improve the properties of the film in this study. This study aims to develop a smart bilayer alginate/agar film containing anthocyanin and catechin–lysozyme film and study the effect of CNs on film’s properties. In addition, application of the developed smart film for shrimp freshness monitoring was also investigated.

## 2. Materials and Methods

### 2.1. Materials

Dried butterfly pea (*Clitoria ternatea*) flowers were purchased from a local market in Chiang Rai, Thailand. Sodium alginate was obtained from Union Science Co., Ltd. (Chaing Mai, Thailand). Agar was purchased from Krungthepchemi Co., Ltd. (Bangkok, Thailand). Catechin hydrate (C1251) and lysozyme from chicken egg white (62971) were purchased from Sigma–Aldrich (St. Louis, MO, USA). *Escherichia coli* TISTR 527 and Staphylococcus aureus TISTR 746 were received from the Biological Laboratory (Mae Fah Luang University, Chiang Rai, Thailand). All other chemicals employed in this study were of analytical grade.

### 2.2. Butterfly Pea Anthocyanin Extraction

The butterfly pea anthocyanin extract (BAE) was prepared using a modified version of the method of Sai-Ut, et al. [40]. In brief, the dried butterfly pea flowers were ground and mixed with 70% (*v*/*v*) ethanol at a 1:40 (g/mL) ratio and then kept in the dark at 4 °C for 18 h. The extracted solution was filtered and then concentrated at 40 °C. Finally, the concentrated extract was freeze-dried (Delta-2-24/LSC plus, Martin Christ, Osterode am Harz, Germany) for 24 h. The BAE powder was stored at −20 °C until used.

### 2.3. Fabrication of Smart Bilayer Film

First, an active layer of film-forming solution (FFS) was prepared with 2% (*w*/*v*) of agar in distilled water and glycerol 50% (*w*/*w*) of based film. The FFS was stirred at 95 °C for 2 h. FFS was then cooled to 65 °C and 0.5% (*w*/*v*) of catechin–lysozyme (ratio 1:1) was added [41]. The FFS was continuously stirred for 30 min. Second, an indicator layer of FFS was prepared using 1.5% (*w*/*v*) of sodium alginate and 50% (*w*/*w*) of based-film glycerol in 100 mL of distilled water. BAE was used as a color indicator at concentrations of 0.5% (*w*/*v*). Following that, corncob cellulose nanosphere (CN) obtained from our previous study (Romruen, Kaewprachu, Karbowiak and Rawdkuen [39]) was added at various concentrations (0, 5, 10, 20, and 30% *w*/*w* SA-based film). The FFS was continuously agitated for 30 min and ultrasonically for 10 min. Finally, active FFS (5 g) was cast onto a silicone plate (5 × 5 cm) and dried at room temperature for 18 h. The indicator FFS (6 g) was then spread on top and allowed to dry at room temperature for 24 h. Before analysis, the dried film was stored in an environmental chamber at 25 ± 0.5 °C and 50 ± 5% RH for 24 h.

### 2.4. Smart Bilayer Film Characterization

#### 2.4.1. Thickness

The thickness of the film was measured using a thickness gauge (C112XBS, Mitutoyo Corp., Kawasaki, Japan) at nine random positions [42].

#### 2.4.2. Appearance and Color

The film’s appearance was recorded by a digital camera (Sony α6000, Sony Thai Co. Ltd., Bangkok, Thailand). The color of the films was determined using a handheld chroma meter (Konica Minolta Sensing Americas Inc., Williams Drive Ramsey, NJ, USA) [43].

#### 2.4.3. Light Transmission and Transparency

The light transmission and transparency properties of developed films were investigated using the method of Jongjareonrak, et al. [44]. The test was performed at wavelengths ranging from 200 to 800 nm, using a UV–Visible spectrophotometer (G105 UV-VIS, Thermo Scientific Inc., Waltham, NJ, USA). Equation (1) was used to calculate the film’s transparency:

(1) $$Transparency=\frac{-logT_{600}}{X}$$

where *T*_600_ is the transmittance at 600 nm and *X* is the thickness of the film (mm).

#### 2.4.4. Morphology of Film

The microstructure of the developed film was investigated using a field emission scanning electron microscope—FESEM (TESCAN MIRA, TESCAN, Brno, Czech Republic)—at a 10 kV acceleration voltage. The image was captured using a magnification of 500× for the upper surface and 1000× for the cross section.

#### 2.4.5. Mechanical Properties

The mechanical properties, including tensile strength (TS) and elongation at break (EAB), of the developed film were calculated using a Universal Testing Machine (Lloyd Instrument, Hampshire, UK). The tests was performed with a 100 N load cell, and ten samples (2 × 5 cm) with a 3 cm grip length were used to measure with the cross-head speed at 30 mm/min [45].

#### 2.4.6. Water Vapor Permeability (WVP)

The WVP of the smart bilayer film was determined using a modified version of the method of ASTM [46]. The films were sealed onto a permeation cup containing silica gel (0% RH). These cups were then placed in an environmental chamber with 50% RH at 25 °C and weighed at 1 h intervals for 8 h, after which the films’ WVP was estimated using Equation (2) [47]:

(2) $$WVP=\frac{W\times X}{A\times t\times \left(P_{2}-P_{1}\right)}$$

where *W* is the cup’s weight gain (g); *X* is the film’s thickness (m); *A* is the film’s area (m^2^); *t* is the gaining time (s); and (*P*_2_ − *P*_1_)^−1^ is the vapor pressure difference throughout the film (Pa). The WVP was represented in units of g m s^−1^ m^−2^ Pa^−1^.

#### 2.4.7. Film Solubility

The water solubility of the developed film was determined using the method published by Gennadios, et al. [48]. A 2 × 2 cm dried film sample was weighed and dissolved into 10 mL of distilled water. Afterwards, it was shaken at 250 rpm in a shaker (Helidolth Inkubator 10000, Schwabach, Germany) at 25 °C for 24 h. After 20 min of centrifugation at 3000× *g*, 25 °C, and undissolved particles were recovered. The pellet was weighed after drying at 70 °C for 24 h. The solubility of the film was computed using Equation (3):

(3) $$Filmsolubility\left(\%\right)=\frac{W_{0}-W_{f}}{W_{0}}\times 100$$

where *W*_0_ is the dry matter weight of the film and *W_f_* is the weight of the dry film residue that had not been dissolved.

#### 2.4.8. pH Sensitivity

The film’s pH sensitivity was assessed using a modified version of the method of Pereira Jr, et al. [49]. Individual 2 × 2 cm pieces of film were immersed in different pH buffer solutions (pH 2 to 12). The appearance changes of the films were photographed by a digital camera after 5 min (Sony α6000, Sony Thai Co. Ltd., Bangkok, Thailand).

#### 2.4.9. Response to Volatile Ammonia and Acetic Acid

The colorimetric reaction was evaluated in response to two pH stimuli solutions: acetic acid (50%, *v*/*v*) and ammonia solution (0.1 M). Each test tube (16 × 150 mm) was filled with 5 mL of stimulus solution before the film being tested was wrapped over the tube mouth. A digital camera (Sony 6000, Sony Thai Co. Ltd., Bangkok, Thailand) was used to record the color shifts of the film over time.

#### 2.4.10. Fourier Transform Infrared Spectroscopy (FTIR)

All films were clamped onto the FT-IR spectrometer mount for FTIR spectra analysis. The films’ infrared spectra were captured at 25 °C using a Fourier Transform Infrared Spectrometer (Raman Spectrometer) (Thermo Fisher Scientific Inc., Waltham, MA, USA) with 64 scans and a 4 cm^−1^ resolution [50].

#### 2.4.11. Thermal Stability

Thermo-gravimetric analysis (TGA) and differential scanning calorimetry were conducted in order to evaluate the thermal stability of the smart bilayer films (DSC). The thermal stability of the films was measured using a thermogravimetric analyzer (Mettler Toledo, Model 851e, Schwerzen-bach, Switzerland) according to the method of Kaewprachu, et al. [51]. Experiments were conducted at 25–700 °C with a heating rate of 10 °C/min in a nitrogen atmosphere (20 mL/min). As stated by Pelissari, et al. [52], the thermal properties of the film were examined using a differential scanning calorimeter (DSC; TA-Instruments, model 2920, New Castle, PA, USA). This test was conducted in a nitrogen atmosphere (20 mL/min) with a temperature scan from 60 to 150 °C at a rate of 10 °C/min.

#### 2.4.12. Bioactive Compounds and Antioxidant Properties

The film’s bioactive compound and antioxidant properties were determined according to the methods of Tongnuanchan, et al. [53]. The film (30 mg) was extracted using distilled water at 250 rpm for 3 h. The supernatant was utilized to assess the total anthocyanin content (TAC), total phenolic content (TPC), ferric-reducing antioxidant power (FRAP), and DPPH radical scavenging activity. According to the methods of Goodarzi, et al. [54], the pH differential method was used to determine the TAC. At 510 and 700 nm, the absorbance of supernatant by the pH 1.0 buffer and pH 4.5 buffer was determined. Calculated as cyanidin-3-glucoside with an extinction value of 26,900 and a molecular weight of 484.83 g mol^−1^. Folin–Ciocalteu assay was utilized to determine TPC. The results are reported in terms of mg GAE/g dry film. For FRAP, film extract solution was combined with FRAP solution and incubated at 37 °C for 30 min, and then the absorbance at 593 nm was measured. The reducing power was expressed as μM ferrous sulfate/g dried film. By mixing film extract solution with methanolic DPPH solution, the absorbance at 517 nm was determined for DPPH. The DPPH activity was measured and reported in terms of mM Trolox/g of dried film. 

#### 2.4.13. Antimicrobial Activity of the Film

The antimicrobial activity of films was determined following the methods of Rawdkuen, Suthiluk, Kamhangwong and Benjakul [41]. The films were evaluated for their ability to inhibit Gram-negative (Escherichia coli) and Gram-positive (Staphylococcus aureus) bacterial strains using the agar disc diffusion method. A bacterial strain concentration of 10^8^ CFU/mL was employed. The films were cut into 5 mm circles, sterilized under UV irradiation for 30 min, and then used to place on surface of agar with the test culture. The test agar plates were incubated at 37 °C for 18 h. The inhibition zone formed around the film disc was utilized to determine the level of antimicrobial activity.

### 2.5. Application of Smart Bilayer Film for Monitoring Shrimp Freshness

The efficacy of smart bilayer films was tested in order to monitor the freshness of shrimp. Two shrimps of similar size were placed in a circular PP tray, and the smart bilayer film (2 × 3 cm) was affixed inside a PVC film wrap, tightly sealed, and stored at 25 °C for 24 h. Changes to the pH and color of the film were observed.

### 2.6. Statistical Analyses

In order to evaluate the analysis of variance, SPSS software (SPSS for Windows version 26.0, SPSS Inc., Chicago, IL, USA) was used to conduct statistical analysis (ANOVA). At a confidence level of 95%, Duncan’s multiple range tests were used to identify the significance of difference between samples.

## 3. Results and Discussion

### 3.1. Thickness

The thickness of the smart bilayer films integrated with corncob cellulose nano-spheres (CNs) at various concentrations are shown in Table 1. The thickness of the films was 0.082, 0.084, 0.091, 0.097, and 0.108 mm for 0% CNs, 5% CNs, 10% CNs, 20% CNs, and 30% CNs, respectively. As the amount of CNs increased, the thickness of the smart bilayer film increased drastically (*p* < 0.05). This increase in thickness may affect other properties of the developed film, especially the mechanical and barrier properties. The increased thickness might be characterized by accumulating a dense network between the sodium alginate matrix (upper layer) and the CNs [55]. These findings were consistent with Salim, Abdellaoui, Ait Benhamou, Ablouh, El Achaby and Kassab [36], who reported that the thickness of chitosan films increased as the concentration of cellulose nanocrystals in pea pod waste increased. Shankar and Rhim [56] also found that increasing the concentration of nanocellulose thickened the agar film. The solid content is primarily responsible for the rise in film thickness.

### 3.2. Mechanical Properties

The mechanical characteristics of smart bilayer films with various concentrations of CNs are shown in Table 1. After the addition of CNs, the mechanical characteristics of the smart alginate/agar bilayer film were altered dramatically, and these qualities were impacted by the amount of CNs added. The film’s tensile strength (TS) increased noticeably (*p* < 0.05). On the other hand, incorporating CNs reduced the film’s flexibility (elongation at break: EAB), inversely proportional to the TS direction. Similar findings were reported by Roy, Kim and Rhim [35], who found that the incorporation of CNs obtained from onion peel significantly improved the mechanical strength of a CMC/agar-based film. As the concentration of CNs rose, the TS of the composite films gradually increased [34]. As nanocellulose has a strong tendency to self-aggregate, the major point of generating nanocomposites with high mechanical performance is the interface compatibility between the filler and the polymer matrix [57]. The CNs used in this study were 2–44 nm in size, which is considered a small nanoparticle with a high self-aggregate. The EAB decreased with increasing CNs concentration (from 61.63% for the neat smart bilayer film to 40.44% for the film with 30% CNs), corresponding to a 34.38% decrease. These findings are consistent with those of Salim, Abdellaoui, Ait Benhamou, Ablouh, El Achaby and Kassab [36], who discovered that the addition of 10% nanocellulose was reduced the EAB value of chitosan film by 37%. The strong interfacial adhesion between the polymer matrix and the CNs can explain this performance, as it limits the matrix motion. Some authors have reported that adding CNs reduces the EAB values of polymer films [58,59]. Aside from interfacial interaction/adhesion, the nanoparticle size influences interfacial/interphase properties [60]. Large nanoparticles exhibit poor interfacial/interphase characteristics and tensile strength even at high interphase thickness, highlighting the importance of particle size.

### 3.3. Water Vapor Permeability (WVP)

The addition of CNs did not significantly affect the WVP of smart bilayer film (*p* > 0.05) (Table 1). The WVP of the developed smart bilayer films with and without CNs ranged from 2.11–2.68 × 10^−10^ g m m^−2^ s^−1^ Pa^−1^. The WVP value of smart bilayer film decreased when integrated with 5% and 10% CNs, but it increased when the CN concentrations were increased to 20% and 30%. However, it was equivalent to the WVP value of CNs-free smart bilayer films. These results were consistent with those of Oun and Rhim [61], who observed that the WVP of CMC film was reduced after a small amount of cellulose nanofiber (CNF) was applied and then steadily increased in conjunction with the CNF concentration. The composite film’s CNs operate as an impermeable barrier to water vapor diffusion, resulting in a more tortuous channel for water vapor diffusion and a lower WVP [62]. Meanwhile, the rise in WVP at 20% and 30% CNs could be related to the discontinuous structure between the CNs and polymer matrix caused by CNs agglomeration at higher concentrations [63], as evidenced by FESEM results.

### 3.4. Film Solubility

The solubility properties of smart bilayer film reinforced with CNs are presented in Table 1. The inclusion of CNs influenced the solubility of smart alginate/agar bilayer films (*p* < 0.05). The solubility of the alginate/agar bilayer film decreased from 58.65% for 0% CNs smart bilayer film to 53.87% for 10% CNs film, which increased with further CNs inclusion. This result indicates that it reached maximum water resistance when 10% CNs was added to the alginate/agar bilayer composite film.

### 3.5. Film Appearance and Color

The inclusion of CNs influenced the appearance and surface color of the smart bi-layer film, as shown in Table 2. All film samples exhibited a blue color, which is representative of the color of BAE. Delphinidin is the primary anthocyanin responsible for the deep blue to purple color of butterfly pea flower [64]. The surface of the smart bilayer film was glossy (without CNs) and matte (with CNs). After adding CNs, the lightness (L*) of the smart bilayer films increased significantly (*p* < 0.05). The increase in film lightness could be attributed to the white color of the CNs powder. Alternatively, the increasing concentration of CNs decreased the greenness (−a*) and blueness (−b*) values of the smart bilayer film (*p* < 0.05). These results indicate that the inclusion of CNs affected the smart bilayer film’s lightness and greenness while not affecting its blueness.

### 3.6. Light Transmission and Transparency

**Figure 1:**
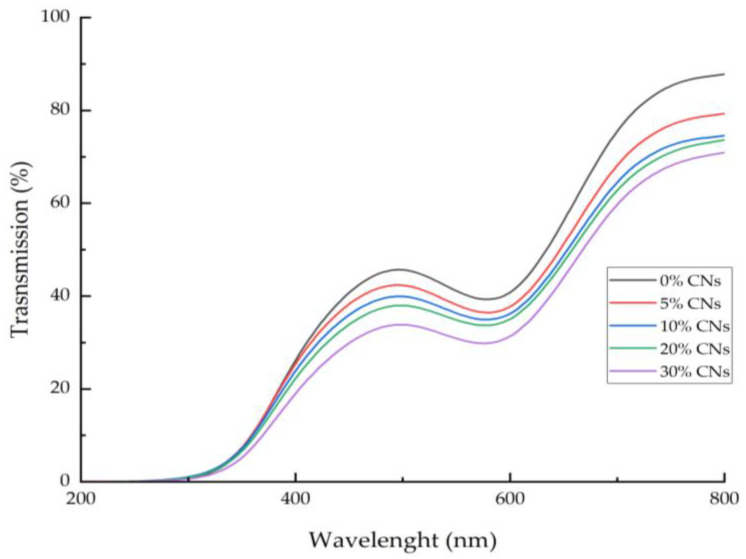
Light transmission of smart bilayer films incorporating different concentrations of cellulose nanosphere (CNs).

The light transmission of a smart bilayer film with various concentrations of CNs incorporation is shown in Figure 1. Smart bilayer films had 0.01 to 0.06% transmission for UV light (200–280 nm), whereas those with and without CNs had transmissions of 1.78 to 87.78% and 1.55 to 70.92%, respectively, for visible light (350–800 nm). As a result of the incorporation of CNs into the smart bilayer film, visible light transmission was diminished. In addition, no UV light transmission was detected in any of the smart bilayer films, regardless of whether or not CNs were present. This consequence could be attributed to the presence of BAE in all of the film formulas. Polyphenol aromatic rings can absorb UV light [65]. The CNs-free film had a light transmission value higher than 80% at 700–800 nm, but the films with CNs added had light transmission values of less than 80% in the visible light spectrum, regardless of concentration. In the visible light spectrum, the film was transparent to the human eye and had a light transmission value greater than 80% [66]. As CNs concentration increased, the transparency of the smart bilayer film reduced dramatically (*p* < 0.05) (Table 2). All of the smart bilayer film samples were proven to be more effective at blocking UV light transmission. Consequently, the smart bilayer film can reduce lipid oxidation in food packing, particularly for high-lipid meals.

### 3.7. Morphology of Film

The microstructures of smart bilayer films with different CN levels are depicted in Table 3. The CNs-free smart bilayer film had a smooth surface with no porous structures, while the CNs-containing films had a rough surface without a porous structure, indicating that the CNs particle films were well dispersed in the polymer matrix, though the degree of roughness of the film surface was dependent upon the CNs concentration. This is primarily due to CNs agglomeration in the polymer matrix at high concentrations [67]. Micrographs show that the laminated layers (sodium alginate [SA] layers) were tightly bound with the lower layer (agar [AG] layer) films due to surface interaction. Due to associative interactions, phase separation could not be detected in all film samples, indicating high compatibility and homogeneity between SA and AG layers [68].

### 3.8. pH Sensitivity

The response of the BAE and smart bilayer films with different concentrations of CNs to the pH buffer is displayed in Table 4. When the pH was raised from 2 to 12, BAE changed from pink to greenish yellow. The BAE was pink when the level of acidity was high (pH 2), and it turned to purple (pH 3), purplish-blue (pH 4–5), and blue (pH 7) as the level changed. At pH levels ranging from 9 to 12, the color of the BAE shifted from blue to green. Rawdkuen, Faseha, Benjakul and Kaewprachu [12] also reported that butterfly pea anthocyanin extracts exhibited the same color changes and were successfully used as a pH indicator in gelatin film. The color of the developed smart bilayer film changed from purple at pH 2–3 to purplish-blue at pH 4–5, blue at pH 6, bluish-green at pH 7–8, green at pH 9–10, and greenish-yellow at pH 11–12. Although the color change shown by the film depends upon the pH levels, the results were in line with the color change of BAE at the same pH values. The color change in response to pH levels of all smart bilayer films is the same color, indicating that the addition of CNs did not affect the pH sensitivity of the smart bilayer film. These results indicate that the color change of a smart bilayer film containing all concentrations of CNs in acidic and alkaline solutions remains visible to the naked eye.

### 3.9. Response to Volatile Ammonia and Acetic Acid

In order to evaluate the responsiveness of smart bilayer films to alkaline and acid gas, a colorimetric analysis of volatile ammonia and acetic acid was carried out. As seen in Table 5, the film was colorimetrically sensitive to volatile ammonia and acetic acid. After exposure to volatile ammonia (0.1M) for 90 min, the smart bilayer film displayed a series of color changes: blue–bluish-green–light green–green for all CNs concentrations. Within 90 min of exposure to acetic acid (50% *v*/*v*), the film displayed a series of color changes: blue–bluish-violet–purple. After being exposed to ammonia and acetic acid, all film samples exhibited the same pattern of color change. However, the film with 5% CNs presented a lighter color and was therefore more difficult to distinguish than other film samples. These results revealed that the developed films both with and without CNs are colorimetrically responsive to surrounding acidic and alkaline volatile variations, which means that they could be applied to monitor food freshness or the stage of fermentation.

### 3.10. FTIR

**Figure 2:**
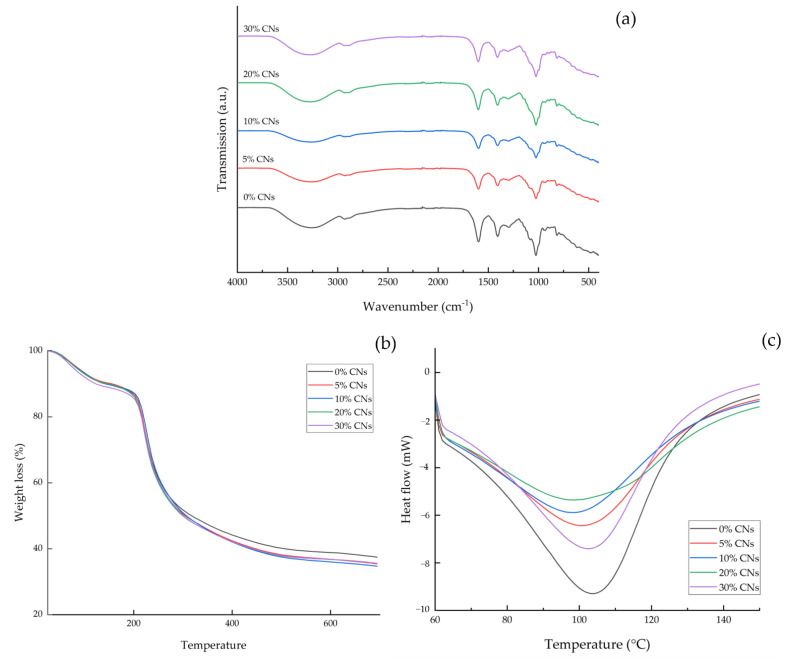
FTIR spectra (**a**), TGA (**b**), and DSC thermograms (**c**) of smart bilayer films incorporating different concentrations of cellulose nanosphere (CNs).

The FTIR-evaluated differences between the chemical structures of smart bilayer films are depicted in Figure 2a. All of the film samples displayed a dominant band at 3250 cm^−1^ and 2925 cm^−1^, pointing to the O-H and C-H stretching vibrations, which corresponds to the aliphatic moieties in polysaccharides [69]. The peaks at 1407 cm^−1^ and 1293 cm^−1^ correspond to the O-H and C-H expansion and sway vibration absorption bands, respectively. Asymmetric and symmetric COO stretching vibrations were assigned to the absorption bands at 1597 cm^−1^ [70]. All of the film samples observed a peak at 2930 cm^−1^, corresponding to C-H aliphatic stretching vibration [71]. The presence of C-H aliphatic stretching vibration represents the phenolic compounds that may have resulted from the incorporation of BAE and catechin. All of the samples had similar diffraction spectra, indicating that the addition of CNs at any concentration did not affect the functional group of the smart alginate/agar bilayer film.

### 3.11. Thermal Stability

The thermal stability of smart bilayer/CNs composite films is shown in Figure 2b. The weight-loss regions in the TGA of smart bilayer films were distinct. The initial weight loss of the smart bilayer film occurred at a temperature between 40 and 120 °C, corresponding to water and volatile compound evaporation and accounting for 9–10% of the weight loss [72]. The second degradation occurred at temperatures ranging from 160 to 300 °C, caused by thermal degradation of the biopolymer chain. Up to the second stage of thermal deterioration, CNs-containing films had slightly higher thermal stability than 0% CNs smart bilayer films, but this difference was not statistically significant. The higher thermal stability of CNs-containing films was associated with the presence of crystalline-form CNs. The CNs composite films had considerably lower residue left after final thermal decomposition at 700 °C than the 0% CNs smart bilayer film. The char of the 0% CNs at 700 °C was 37.33%, but it decreased to 34.55–35.48% depending on the CNs concentration. The findings were consistent with those of Reddy and Rhim [73], who reported that adding mulberry pulp CNC resulted in less residue at 600 °C than neat agar film.

The calorimetric thermogram for all film samples is depicted in Figure 2c. The endothermic peaks that occur in DSC curves are typically associated with the breaking of hydrogen bonds [74]. The DSC thermogram of the smart bilayer film and the CNs composite film samples revealed that the transitions occur over a wide temperature range. The addition of CNs did not affect the melting temperature of the smart bilayer film. After the addition of CNs, the melting temperature of the smart bilayer film was reduced from 104.2 °C to 98.83–103.11 °C. This result agreed with Chen, et al. [75], who found that the melting temperature decreases as the nanocellulose concentration increases.

### 3.12. Bioactive Compounds and Antioxidant Properties

The antioxidant properties are the common essential functional role of the active films. They have the ability to effectively prevent oxidative deterioration in packaged foods. Since CNs have no superior antioxidant properties, their incorporation alone does not affect the antioxidant properties of the film. As a result, CNs are frequently used in films as a synergist with the polymer matrix or other active ingredient additives in order to enhance the antioxidant and antibacterial properties [76]. The bioactive compounds and antioxidant properties of smart bilayer films with varying concentrations of CNs are presented in Table 6. The inclusion of CNs had no significant effect on the total anthocyanin and TPC of smart bilayer film (*p* > 0.05). The anthocyanin content in smart bilayer films ranges from 2.19 to 2.42 mg/100 g db. The TPC of smart bilayer film ranges between 46.40 and 51.88 mg GAE/g db. The increase in CNs content had a minor effect on the TPC of smart bilayer film. The presence of the phenolic compound in the smart bilayer film resulted from the BAE and catechin in the film formulation. Catechins have been reported to be excellent antioxidant agents [51]. Kaewprachu, Osako, Rungraeng and Rawdkuen [51] also reported that films incorporating catechin–Kradon extract had higher antioxidant activity than exclusively Kradon extract-added fish myofibrillar protein films. All of the film samples had the same concentration of BAE and catechin, but the addition of CNs increased the total weight of the film-forming solution, causing the bioactive compounds’ intensity to decrease.

The antioxidant properties of smart bilayer film samples, including ferric-reducing antioxidant power (FRAP) and DPPH radical scavenging activity, are shown in Table 6. All of the film samples had antioxidant properties ranging from 518.79 to 575.09 mM Fe (II)/g db. for FRAP and 628.25 to 1033.87 mM Trolox/g db. for DPPH. The inclusion of CNs had the effect of decreasing the FRAP values of the film (*p* < 0.05). Conversely, as the concentration of CNs increased, the DPPH values of the smart bilayer film increased (*p* < 0.05). Furthermore, CNs can improve antioxidant properties by interacting directly with the film matrix. Mujtaba, Akyuz, Koc, Kaya, Ilk, Cansaran-Duman, Martinez, Cakmak, Labidi and Boufi [55] revealed that the incorporation of cellulose nanofiber (CNF) greatly enhanced the antioxidant and antibacterial properties of mucilage films by enhancing their releasing behavior and solubility. Nanocellulose, as a controlled release agent and stabilizer, is an excellent filler for enhancing the antioxidant and antibacterial characteristics of films. These findings imply that the developed smart bilayer films, both with and without CNs, have excellent antioxidant properties and high phenolic contents. The smart bilayer film with high antioxidant activity can be used for active packaging in order to preserve food quality and extend food shelf life by preventing food oxidation.

### 3.13. Antimicrobial Activity

The antibacterial activity against *Escherichia coli* and *Staphylococcus aureus* as measured by the diameters of the clear zones of smart bilayer films is presented in Table 6. All of the smart bilayer films displayed 10.00–11.67 mm diameter inhibition zones against *S. aureus* and 10.67–11.67 mm diameter inhibition zones against *E. coli*. All of the film samples exhibit outstanding antibacterial activity against Gram-positive and Gram-negative bacteria, regardless of the CNs concentration (*p* > 0.05). Due to CNs having no obvious antimicrobial activity, their incorporation into the film does not affect its antimicrobial properties. The antimicrobial properties of smart bilayer film are attributed to the presence of catechin–lysozyme in the film formulation. For all film samples, the same concentrations of catechin–lysozyme were used. As a result, there was no difference in the antimicrobial activity of all smart bilayer film samples. The mechanical and antimicrobial properties of lysozyme consist of hydrolysis of peptidoglycan layers in bacteria’s cell wall and membrane disruption [77]. Rawdkuen, Suthiluk, Kamhangwong and Benjakul [41] reported the antimicrobial activity of gelatin film incorporating catechin–lysozyme at different concentrations (0.125 to 0.5%, *w*/*v*). The added catechin–lysozyme film inhibited all of the tested microorganisms: *S. cerevisiae*, *S. aureus*, *E. coli*, and *L. innocua*. Another study indicated that lysozyme combined with nanocellulose had the strongest antibacterial effects against *S. aureus* and *E. coli* [78]. Tea polyphenol has excellent antibacterial properties as a natural active substance with a high antioxidant capacity [79]. According to Jamróz, et al. [80], incorporating green and black tea extracts into the furcellaran–gelatin film can increase its antibacterial efficacy against *S*. *aureus* and *E*. *coli*. Consequently, smart bilayer films containing all concentrations of CNs inhibit the growth of microorganisms in food during storage, resulting in the prolonged shelf-life of food.

### 3.14. Application of Smart Bilayer Film for Monitoring Shrimp Freshness

**Figure 3:**
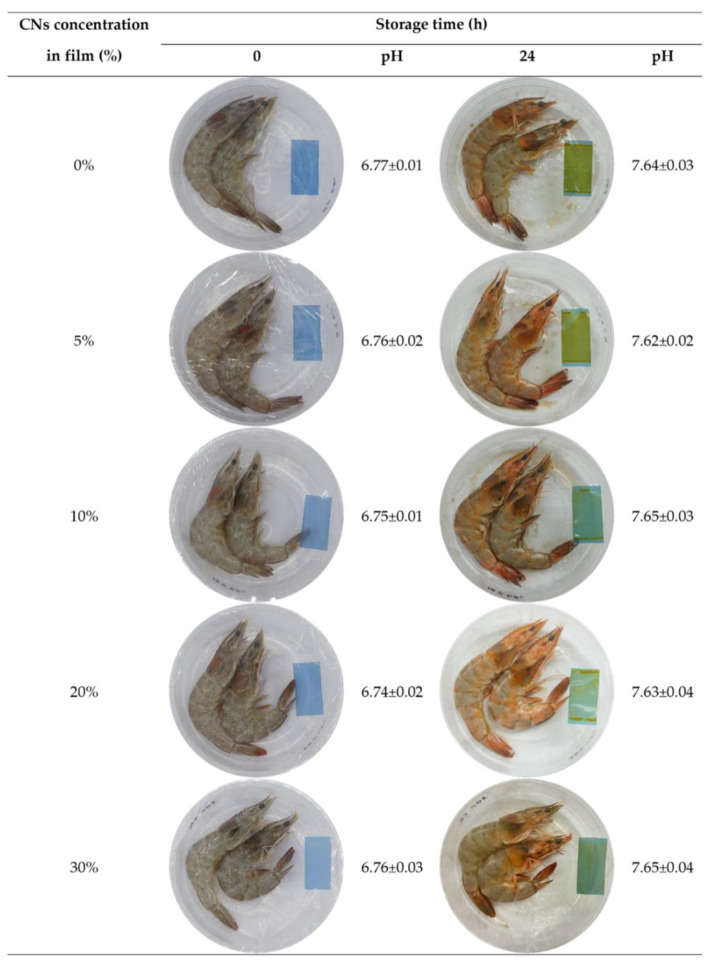
Application of a cellulose nanosphere (CNs)-containing smart bilayer film for monitoring changes in shrimp quality at 25 °C.

Due to high concentrations of total volatile basic nitrogen (TVB-N), spoilage of meat, fish, and poultry items causes an increase in pH from 5.8 to 7.8 [81]. Therefore, shrimp was used to evaluate the use of smart bilayer film containing various concentrations of CNs for monitoring freshness at 25 °C (accelerated conditions). After 24 h of storage, as a pH-detecting indication (untouched by the shrimp), all of the film samples exhibited consistent color changes (Figure 3). In addition, after 24 h, the film’s color changed from blue to green, and the pH changed from 6.74 to 7.65 (Figure 3). Consequently, smart bilayer films, both with and without CNs, could be used as intelligent food packaging films in order to monitor the freshness of seafood or other fresh meat products through direct visual inspection.

## 4. Conclusions

The addition of butterfly pea anthocyanin extract and catechin–lysozyme to film formulations resulted in the successful preparation of a smart bilayer film. The smart bilayer film has a blue film color and has the ability to change colors depending on the pH level. Furthermore, it has the potential to change color when exposed to volatile ammonia and acid. The smart bilayer film is highly antioxidant and antimicrobial. Corncob cellulose nanospheres (CNs) can improve the mechanical properties of smart bilayer films without affecting their chemical properties. The smart bilayer film, containing all concentrations of CNs, was successfully used to monitor the freshness of the shrimp. After the shrimp spoiled, the color of the smart bilayer film changed from blue to green. These findings imply that smart bilayer films with and without CNs can be used in food packaging systems to monitor spoilage without causing damage and to extend the shelf life of food.

## Figures and Tables

**Table 1:** Physical and mechanical properties of smart bilayer film incorporated with different concentrations of cellulose nanosphere (CNs).

CNs Concentration in Film (%)	Thickness (mm)	TS * (MPa)	EAB * (%)	WVP (×10^−10^ g m/m^2^ s Pa)	Film Solubility (%)
0	0.082 ± 0.002 ^d^	16.11 ± 1.32 ^c^	61.63 ± 3.30 ^a^	2.27 ± 0.06 ^a^	58.65 ± 1.06 ^b^
5	0.084 ± 0.002 ^d^	18.26 ± 2.01 ^b^	50.39 ± 7.19 ^b^	2.11 ± 0.10 ^a^	56.52 ± 1.32 ^bc^
10	0.091 ± 0.001 ^c^	18.63 ± 1.53 ^b^	46.27 ± 4.97 ^bc^	2.22 ± 0.05 ^a^	53.87 ± 2.43 ^c^
20	0.097 ± 0.001 ^b^	19.02 ± 1.20 ^ab^	41.86 ± 5.69 ^cd^	2.34 ± 0.11 ^a^	61.93 ± 1.72 ^a^
30	0.108 ± 0.002 ^a^	20.51 ± 2.06 ^a^	40.44 ± 4.17 ^d^	2.68 ± 0.08 ^a^	63.43 ± 0.97 ^a^

Values are given as mean ± SD, *n* = 3. * *n* = 10 for TS and EAB. Different superscripts in each column are significantly different (*p* < 0.05).

**Table 2:** Appearance, color, and transparency of smart bilayer film incorporated with different concentrations of cellulose nanosphere (CNs).

CNs Concentration in Film (%)	Appearance	L*	a*	b*	Transparency (%)
0	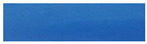	44.97 ± 1.19 ^c^	−10.33 ± 0.63 ^c^	−34.17 ± 0.66 ^b^	2.50 ± 0.01 ^a^
5	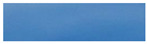	48.28 ± 0.23 ^b^	−9.28 ± 0.45 ^b^	−30.02 ± 0.64 ^a^	2.44 ± 0.02 ^b^
10	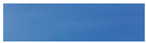	49.01 ± 1.06 ^ab^	−8.67 ± 0.05 ^b^	−29.89 ± 0.59 ^a^	2.41 ± 0.05 ^bc^
20	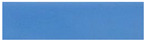	49.65 ± 0.81 ^ab^	−8.59 ± 0.19 ^b^	−29.65 ± 0.26 ^a^	2.39 ± 0.01 ^c^
30	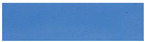	50.07 ± 0.17 ^a^	−7.57 ± 0.24 ^a^	−29.51 ± 0.41 ^a^	2.27 ± 0.01 ^d^

Values are given as mean ± SD, *n* = 3. Different superscripts in each column are significantly different (*p* < 0.05).

**Table 3:** Surface and cross-section morphology of smart bilayer films incorporating different concentrations of cellulose nanosphere (CNs).

CNs Concentration in Film (%)	Upper Surface	Cross-Section
0	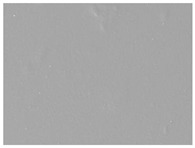	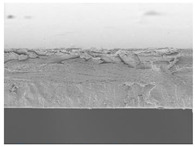
5	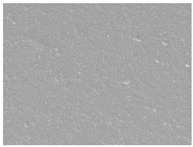	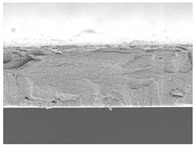
10	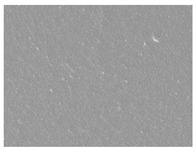	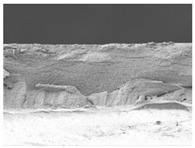
20	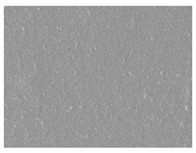	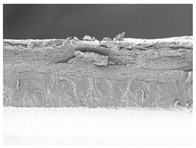
30	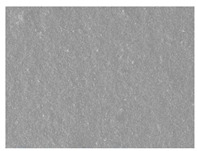	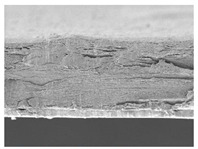

The magnifications used for surface and cross-section images are 500× and 1000×.

**Table 4:** pH sensitivity of BAE and smart bilayer film incorporated with different concentrations of cellulose nanosphere (CNs).

pH	BAE Solution (0.05% *w*/*v*)	Concentration (%) of Cellulose Nanosphere (CNs)				
		0	5	10	20	30
Original	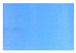	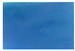	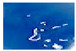	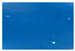	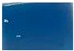	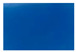
2	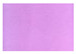	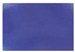	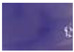	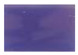	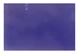	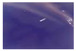
3	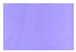	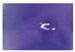	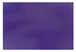	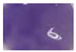	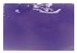	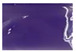
4	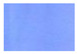	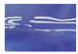	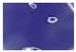	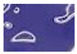	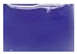	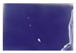
5	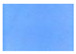	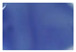	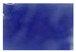	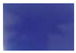	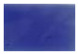	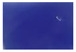
6	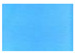	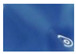	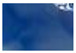	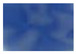	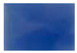	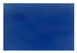
7	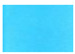	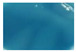	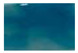	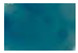	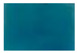	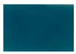
8	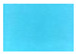	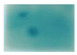	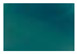	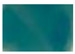	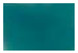	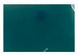
9	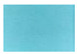	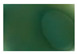	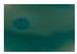	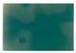	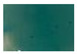	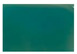
10	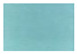	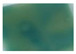	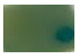	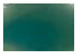	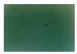	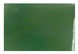
11	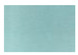	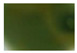	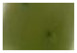	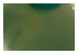	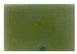	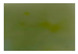
12	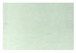	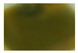	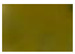	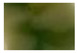	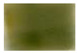	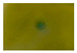

**Table 5:** Response to volatile ammonia and acetic acid of smart bilayer films incorporating different concentrations of cellulose nanosphere (CNs).

CNs Concentration in Film (%)	Exposure Time (min) in 50% Acetic Acid				Initial Film	Exposure Time (min) in 0.1 M Ammonia			
	90	60	30	15		15	30	60	90
0	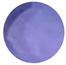	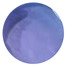	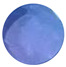	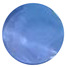	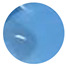	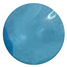	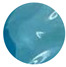	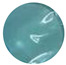	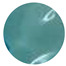
5	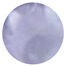	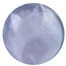	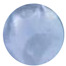	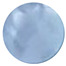	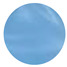	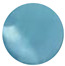	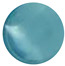	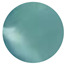	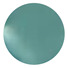
10	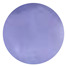	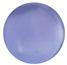	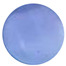	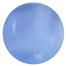	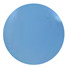	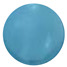	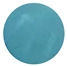	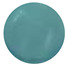	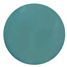
20	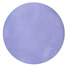	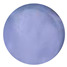	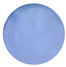	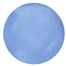	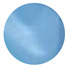	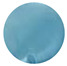	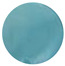	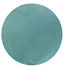	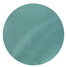
30	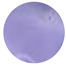	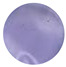	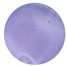	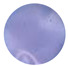	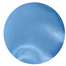	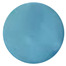	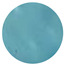	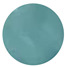	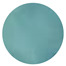

**Table 6:** Antioxidant and antimicrobial properties of smart bilayer films incorporating different concentrations of cellulose nanosphere (CNs).

CNs Concentration in Film (%)	Bioactive Compounds Antioxidant Properties				Antimicrobial Activity (mm)	
	Anthocyanin (mg/100 g)	TPC (mg GAE/g)	FRAP (mM Fe (II)/g)	DPPH (mM Trolox/g)	*E* *. coli*	*S* *. aureus*
0	2.30 ± 0.24 ^a^	46.58 ± 1.14 ^a^	575.09 ± 1.18 ^a^	628.25 ± 8.50 ^c^	11.67 ± 0.58 ^a^	11.67 ± 0.58 ^a^
5	2.33 ± 0.23 ^a^	46.54 ± 1.08 ^a^	551.67 ± 13.97 ^ab^	911.54 ± 51.68 ^b^	10.67 ± 0.58 ^a^	10.83 ± 0.76 ^a^
10	2.19 ± 0.12 ^a^	45.66 ± 1.12 ^a^	525.96 ± 17.23 ^bc^	943.04 ± 35.17 ^b^	11.17 ± 0.29 ^a^	11.67 ± 0.29 ^a^
20	2.42 ± 0.08 ^a^	45.29 ± 0.16 ^a^	522.83 ± 26.47 ^bc^	1020.98 ± 15.39 ^a^	10.67 ± 0.58 ^a^	10.67 ± 0.58 ^a^
30	2.38 ± 0.21 ^a^	45.36 ± 0.08 ^a^	518.79 ± 2.52 ^c^	1033.87 ±9.31 ^a^	10.83 ± 0.29 ^a^	10.00 ± 0.00 ^a^

Values are given as mean ± SD, n = 3. Different superscripts in each column are significantly different (*p* < 0.05).

## Data Availability

The data presented in this study are available on request from the corresponding author.
